# The effect of diluting povidone-iodine on bacterial growth associated with speech

**DOI:** 10.1186/s12886-019-1066-5

**Published:** 2019-02-26

**Authors:** Sivashanth Gnanasekaran, Sophie Rogers, Sanj Wickremasinghe, Sukhpal S. Sandhu

**Affiliations:** Centre For Eye Research Australia, The Royal Victorian Eye and Ear Hospital, University of Melbourne, 32 Gisborne Street, East Melbourne, Victoria 3002 Australia

**Keywords:** Anti-VEGF, Endophthalmitis, Intravitreal injection, Povidone-iodine, Pre-injection antisepsis

## Abstract

**Background:**

Povidone-Iodine (PI) may be diluted when used as an antiseptic prior to an intravitreal injection in an attempt to decrease patient discomfort. This study aims to investigate the effect of diluting povidone-iodine (PI) on bacterial growth from bacterial droplet dispersal associated with speech.

**Methods:**

Participants read a standardised script for 5 min over a blood agar plate positioned at 20 cm in a simulated position of an intravitreal injection procedure. The blood agar plates were subject to a randomised pre-application of 1% PI; 2.5% PI; 5% PI and no pre-application (control). The plates were incubated at 37 °C for 72 h and the number of Colony Forming Units (CFUs) was determined. CFUs were summarised as median and interquartile range (IQR). Wilcoxon rank sum test was used to assess pairwise comparisons of the various PI concentrations to the control group. Any trend across PI concentration was assessed using Kendall’s tau rank correlation.

**Results:**

Twenty-one subjects participated. Control plates had a median growth of 25 CFUs (interquartile range [IQR]:15–40), 1% PI plates had a median growth of 30 CFUs (IQR:15–82), 2.5% PI had a median growth of 18 CFUs (IQR:10–32) and 5% PI had a median growth of 2 CFUs (IQR:0–5). There was significantly less bacterial growth with 5% PI compared to control (*P* < 0.001). Bacterial growth at 2.5% PI and 1% PI did not differ significantly from control. There was a statistically significant trend for decreasing colony count as PI concentration increased (*P* < 0.001).

**Conclusions:**

PI concentrations less than 5% are not effective at reducing bacterial growth from bacterial droplet dispersal associated with speech. When using PI for pre-injection antisepsis, concentrations below 5% should be avoided.

## Background

Intra-ocular drug treatments for a number of diseases of the retina have increased exponentially over the last decade. Intravitreal anti-vascular endothelial growth factor (VEGF) injections are the current standard of care for choroidal neovascularisation (CNV) secondary to age-related macular degeneration (AMD) and retinal vascular disorders, both sight-threatening diabetic macular oedema (DME) and retinal vein occlusion (RVO) with cystoid macular oedema (CME) [1–4].

A rare but potentially blinding complication of any invasive intra-ocular procedure is endophthalmitis. Large studies have found the rate of endophthalmitis following intravitreal injection (IVI) to range from 0.018 to 0.057% [5–8]. These per-injection rates are low but as patients receive regular injections, the additive risk associated with a course of treatment becomes significant. The Comparison of Age-related Macular Degeneration Treatments Trials (CATT), looking at the effectiveness of bevacizumab and ranibizumab in 1185 patients with exudative AMD reported per-patient rates of endophthalmitis of 0.93% over 2 years [9]. *Streptococcus species* has been identified as significant causes of culture-positive endophthalmitis following IVIs [8, 10–12]. However *Streptococcus species* make up only a small portion of the normal conjunctival flora [13, 14]. It is postulated that microbes from the upper respiratory tract (URT) colonise the conjunctiva of the eye and further, that there is an additive effect through droplet spread from the treating doctor, nurse or patient [5]. Inoculation of bacteria is then thought to occur at the time that the needle penetrates the sclera.

Povidone-Iodine (PI) has the strongest evidence for reducing the rate of endophthalmitis post cataract surgery but its evidence in relation to IVIs is limited [15, 16]. Animal models have shown that PI is toxic to the cornea [17–19]. Jiang et al found 0.5% PI and 1% PI to be safer in the rabbit cornea and reasoned they should be preferentially used for preoperative antisepsis [17]. The effect of regular or repeated exposure of PI on the human cornea has not been fully explored but there is some evidence of both increased patient reported symptoms and objective signs of dry eyes in these patients [20]. Furthermore, pre-IVI topical anaesthesia masks corneal epithelial toxicity caused by PI and can generate significant patient discomfort once the anaesthesia wears off. As such, clinicians are anecdotally developing strategies to combat this, as not all are convinced that chlorhexidine is a safe and valid alternative. When concerns for patient discomfort or the concern of an allergic reaction are high enough, PI has not been used and the observed rates of endophthalmitis are much higher [21, 22]. Alternative strategies for asepsis pre-IVI that reduce the concentration of PI include: using a cotton pledget to ‘stain’ the area of conjunctiva at the site of injection, dilution of PI to lower concentrations using the anaesthetic minims vial, or diluting in a syringe with sterile normal saline or sterile water for injection. The clinical safety of this practice in the context of IVIs has not been explored formally but Modjtahedi et al report endophthalmitis in a patient in whom a modified PI prep was used pre-injection [22].

This study aims to investigate if lower concentrations of PI are effective at reducing bacterial growth associated with bacterial dispersal from speech, and to explore if the practice of diluting PI to concentrations lower than 5% represents a viable option for effective IVI antisepsis or if this practice is compromising patient safety.

## Methods

This study was approved by The Royal Victorian Eye and Ear Hospital’s human research ethics committee and involved a series of healthy participants. Participants included staff and students at the Centre For Eye Research Australia (CERA). Once recruited with informed consent, which included a detailed protocol, each participant, along with an assistant entered a room where IVIs are regularly performed. This is in the hospital’s outpatient department, and is identified and secluded as an area where only intravitreal injection treatments are performed. The participant and assistant were gowned and gloved in simulation of IVI conditions. A blood agar test plate was secured to the head of a standard reclining ophthalmologic treatment chair. Participants were positioned such that their lips were approximately 20 cm above the blood agar test plate. The agar plates were 100 × 15 mm (diameter x height) in size and this position was chosen to mimic the proximity of a treating clinician to a patient’s eye during an intravitreal injection procedure. The participant then read out loud a standard script for 5 min. After reading for 5 min the blood agar test plate was removed and the participant rested for 5 min before reading from the script again above a new blood agar test plate. Each participant read over four individual blood agar test plates subject to a pre-application of (1) 1% PI; (2) 2.5% PI; (3) 5% PI; (4) no pre-application. The randomised order in which the plates were presented was generated using Microsoft Excel’s random number generator. The diluted concentrations of PI were generated by drawing up combinations of 5% PI and 0.9% saline into a 2.5 ml syringe. The surface of the agar plate was irrigated with PI in the same way PI would be applied to the ocular surface prior to an intravitreal injection. The agar plates were then inverted to allow the excess PI to drain off. The agar plate sat for 30 s before being placed in front of the study participant to mimic the recommended wait time of 30 s before injection [23]. All blood agar plates were sealed and marked, and taken to St Vincent’s Clinical Microbiology Laboratory, where they were incubated at 37 °C for 72 h. The number of Colony Forming Units (CFUs) was determined using standard laboratory techniques by staff blinded to the plate collection sequence. All statistical analysis was undertaken using Stata IC 12.1 for Windows (StataCorp LP, College Station, TX). CFUs were summarised as median and interquartile range (IQR). Wilcoxon rank sum test was used to assess pairwise comparisons of the various PI concentrations to the control group. Any trend across PI concentration was assessed using Kendall’s tau rank correlation.

## Results

**Fig. 1:**
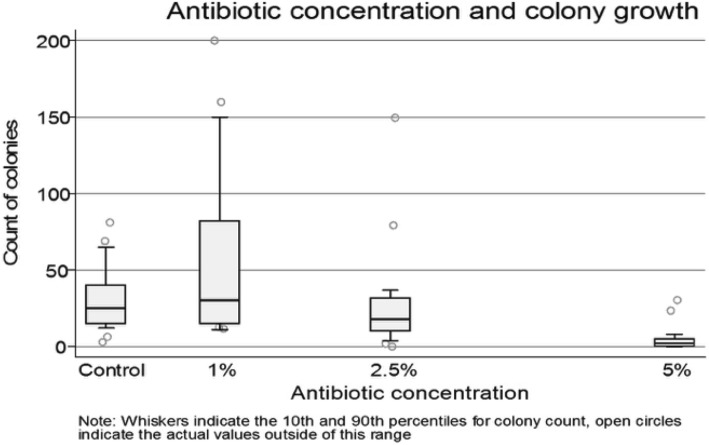
Box plot of CFUs with varying PI concentration. Lower quartile, median and upper quartile are represented by the box. Whiskers indicate the 10th and 90th percentiles for colony count; open circles represent values outside of this range

A total of 21 participants were recruited and provided samples for a total of 84 blood agar plates. There were 6 males and 15 females. The mean age of the participants was 29.7 years (range 21–51). Control plates had a median growth of 25 CFUs (IQR:15–40), 1% PI plates had a median growth of 30 CFUs (IQR:15–82), 2.5% PI had a median growth of 18 CFUs (IQR:10–32) and 5% PI had a median growth of 2 CFUs (IQR:0–5). Figure 1 summarises the bacterial growth associated with the varying PI concentrations. Whilst all control, 1% PI and 2.5% PI plates generated at least 1 CFU of bacterial growth, only 13 of the 21 5% PI plates demonstrated at least 1 bacterial colony of growth. Lowered concentrations of PI were not effective at reducing bacterial growth; there was no statistically significant difference in CFUs between 1% PI and control or between 2.5% PI and control. There was a statistically significant difference in bacterial growth between control and 5% PI plates (Wilcoxon rank sum test *p* < 0.001). Furthermore, there was a statistically significant trend for decreasing colony count as antiseptic concentration increased (Kendall’s tau, *p* < 0.001).

## Discussion

This in-vitro experiment demonstrates that bacterial flora from an IVI administering clinician is potentially dispersed onto an operative field by speech. It also shows that PI is effective at reducing the growth associated with this bacterial droplet dispersal associated with speech. In this study, 5% PI was significantly better than lower concentrations of PI, 1 and 2.5%, at reducing bacterial growth. This finding is consistent with the findings of Doshi et al*,* who found 5% PI to generate lower CFUs than wearing a facemask or no speech [15].

PI has been shown to be toxic to the cornea in animal models. Jiang et al showed concentrations as low as 2.5% PI were toxic to the rabbit’s cornea and that 1% PI and 0.5% PI did not to generate corneal toxicity in the rabbit’s cornea [17]. Similar findings were also made by Lerhaupt and Mauger, who showed a graded increase in corneal endothelial loss with increasing PI concentrations [18]. The cornea is most prone to PI toxicity when the cornea is dry. Aging is also linked to poor tear film production and thus drier eyes [24]. Patients receiving regular intravitreal injections report increased symptoms of dry eyes and have increased objective signs of dry eyes [20]. The cornea of an older patient, with a lid speculum in place, exposed to PI for 30 s significantly increases the chances of corneal toxicity. Given the regularity of IVIs, if lower concentrations of PI provide effective antisepsis, the risk of corneal toxicity could be minimised. Thus, the motivation for assessing whether lower concentrations of PI provide effective antisepsis stems from concerns surrounding patient discomfort and safety. Even with the appropriate use of topical anesthetic, PI use during the IVI procedure generates patient discomfort after the anesthetic wears off, significantly more so in patients with ocular surface disease. Oakley et al investigated pain scores post PI application and post aqueous chlorhexidine application to the ocular surface in healthy subjects and found participants reported significantly higher pain scores in the eyes exposed to PI [25]. No published surveys into IVI procedure technique have questioned ophthalmologists on the practice of using lower PI concentrations for asepsis [26–28]. A recent multi-center study suggests ophthalmologists may be using concentrations as low as 1% PI prior to IVIs [29]. Guidelines recommend a drop of 5–10% of PI, and any survey would be biased by ‘guideline advice’ and ‘true practice’. Waiting 30 s post PI administration has been shown to be most effective at reducing the number of colonies cultured from the conjunctiva [23]. Rates of endophthalmitis are higher when PI is not used pre-injection and some argue that formal allergy testing be considered in patients before PI is avoided as pre-injection antisepsis [21, 22].

In vivo and in vitro studies have differed in their findings on the antiseptic activity of PI at lower concentrations. In vitro, Berkelman RL et al showed greater bactericidal efficacy at concentrations of PI ranging from 0.1 to 1% [30]. Lower concentrations of PI have been shown to have greater concentrations of free iodine which is cytotoxic to cells and leads to cell death [31, 32]. This was reproduced by Roberts et al in a study on the ocular surface of dogs and Grimes SR et al showed in a small study of 22 patients 0.02% PI irrigation was comparable to 5% PI drops [33, 34]. More recently, Peden et al found a lower incidence of endophthalmitis with the use of dilute PI as opposed to 5% PI for pre-injection antisepsis [35]. However, a randomised prospective study found lower concentrations of PI to be less effective than higher concentrations at reducing conjunctival bacterial flora pre-operatively in patients [36]. Our in-vitro study shows 5% to be more effective than lower concentrations at reducing CFUs associated with bacterial dispersal from speech.

This in vitro study could be perceived to have a number of limitations. Whilst there is a small sample size of 21 subjects, the use of a test for trend across all PI concentrations maximised the statistical sample and found statistical significance. Furthermore, a standardised distance of 20 cm was utilised with reading of a standardised script for 5 min. In reality the distance between patient and clinician may vary both within a given IVI episode and between clinicians. The time spent talking will also differ between patients and from clinician to clinician. Assistants will bring an additive effect. The time duration was chosen to maximise the potential bacterial dispersal onto the blood agar plates and by extension assess the effectiveness of PI under a heavy bacterial load. Even under this heavy bacterial load, 5% PI did not generate any growth on 8 of the 21 (38%) plates; supporting the claim that effective pre-injection antisepsis is essential for reducing the risk of endophthalmitis. The methodology of this study stems from similar studies conducted both in the field of ophthalmology and other fields of medicine [15, 37, 38]. Additional limitations could include the blood agar plate having a surface area greater than the ocular surface and its makeup not being an accurate representation of the ocular surface. It is also presumed that bacteria on the blood agar plates correlate with the risk of post-injection endophthalmitis. Finally, we did not to identify the organisms grown on the culture plates and consequently cannot be sure that the CFUs that developed were secondary to the dispersion of URT bacterial species alone. This study also found 1% PI plates to generate greater CFUs than the control plates, though this is not statistically significant. We hypothesise that the moisture the saline could bring to the agar plate may have provided a more favorable medium for bacterial growth than the normal agar plate surface. Both moisture and humidity have been shown to affect colony growth and morphology previously [39, 40]. Future studies should utilise an agar plate pre-exposed to saline as the control.

## Conclusions

In conclusion, PI concentrations lower than 5% are not effective at reducing significant bacterial growth associated with bacterial dispersal from speech. Ophthalmologists should avoid diluting PI to concentrations lower than 5% for pre-injection antisepsis.

## Abbreviations

CFU: Colony forming unit
DME: Diabetic macular oedema
IQR: Interquartile range
IVI: Intravitreal injection
PI: Poviodone-iodine
URT: Upper respiratory tract
